# Cancer immunotherapy in patients with new or recurrent malignancies after liver transplantation

**DOI:** 10.1097/IJ9.0000000000000049

**Published:** 2017-11-16

**Authors:** Mengqi Liu, Wenzhi Guo, Shuijun Zhang

**Affiliations:** a Department of Hepatobiliary and Pancreatic Surgery, The First Affiliated Hospital of Zhengzhou University; b Henan Key Laboratory of Digestive Organ Transplantation, Zhengzhou, Henan, China

**Keywords:** Cancer, Immunotherapy, Liver transplantation, Recurrence

## Abstract

Cancer immunotherapy, as a new treatment modality, has been shown to be effective, especially in metastatic melanoma and lung cancer. Organ transplantation can be a life-saving procedure for patients with end-stage diseases of lung, heart, kidney and liver. While ironically, as improvements in organ transplantation have extended patients’ lives, new or recurrent postsurgical malignancies have become an increasing threat to their long-term survival, especially in patients after liver transplantation due to hepatocellular carcinoma. The feasibility of immunotherapy treatment for such patients is still to be investigated.

Cancer immunotherapy involves redirecting the patient’s own immune system against his or her cancer rather than targeting the cancer itself^1^,^2^. This new treatment modality has been shown to be effective^3^,^4^, especially in metastatic melanoma and lung cancer^5^,^6^. Ironically, as improvements in organ transplantation have extended patients’ lives, new or recurrent postsurgical malignancies have become an increasing threat to their long-term survival. Here we will give a general review of the literature regarding cancer immunotherapy in addition to presenting reviewing 3 case reports of liver transplant patients treated with immunotherapy.

Tumor cells are genetically unstable and often display antigens not found elsewhere in the body, therefore prompting immune reactions. Cancer immunotherapies thus essentially use T cells as an anticancer drug^7^. In theory, if a patient’s T cells are reacting to tumor antigens, this approach can be used for any type of cancer. T cells can be activated mainly through (a) use of checkpoint inhibitors (antibodies directed against immune-regulatory checkpoint molecules expressed on T cells); (b) adoptive transfer of anticancer T cells; and (c) through induction in vivo by vaccination or endogenous delivery of neoantigens subsequent to other anticancer therapies^8^. Among these 3 approaches, the use of checkpoint inhibitors has achieved the most impressive clinical results so far. Immune checkpoints refer to a variety of inhibitory pathways that are crucial for regulating the duration and amplitude of physiological immune responses in peripheral tissues in order to minimize collateral tissue damage^9^. However, if these immune checkpoint pathways are co-opted by cancer cells, which is a hallmark of cancer, it can circumvent immune destruction^10^. The first immune checkpoint inhibitor to be approved by the Food Drug and Administration was ipilimumab in March 25, 2011, a cytotoxic T lymphocyte-associated antigen-4 (CTLA-4) inhibitor. By the end of 2014, nivolumab and pembrolizumab, both programmed death 1 (PD-1) inhibitors, were also approved^11^.

Since the first human liver transplantation (by Thomas Starzl in 1963), liver transplantation is increasingly used to treat end-stage liver diseases, including hepatocellular carcinoma (HCC). An estimated 782,500 new liver cancer cases and 745,500 deaths occurred worldwide during 2012. Although patients who satisfy the Milan Criteria have a 5-year survival >70%^12^,^13^—similar to the prognosis of noncancer patients, and several expanded criteria for HCC beyond the Milan criteria had been proposed, such as the University of California, San Francisco (UCSF) criteria^14^, the “up to 7 criteria”^15^ and the Hangzhou criteria^16^, giving more patients chances for cures—many will develop local or systemic recurrences and metastases, or even new malignant tumors, and ultimately, succumb to their disease^17^,^18^. Furthermore, the suppressed immunity of transplant patients has been associated with a higher incidence of cancer. For example, compared with the age-matched controls, organ transplant recipients (OTRs) have an estimated 3.6-fold increased risk of developing melanoma^19^; as degree of immunosuppression is negatively correlated with survival, prognosis for these patients is also estimated to be worse than for patients with nontransplant associated melanomas^19^. Systemic therapy for new or recurrent tumors, both localized and metastatic, has seen minimal progress over the past 2 decades. Current approaches rely upon cytotoxic chemotherapy combinations aimed at increasing cure rates or achieving palliation and disease control, but these regimens are fraught with short-term and long-term toxicities and outcomes remain suboptimal.

New therapeutic options are urgently needed. Systemic immunotherapies that can provide durable remissions in patients with other malignancies could transform the field. Immunotherapy has become the focus of growing interest, especially after the impressive results of checkpoint blockade inhibitors in malignant melanoma and non–small cell lung cancer, which indicate the therapeutic potential of tumor-specific immune restoration. Initially, solid-OTRs were excluded from clinical trials with cancer immunotherapies because of their use of immunosuppressive agents^20^,^21^. As most immunotherapies for organ transplantation are intended to achieve enough immunosuppression to prevent organ rejection or limit auto-reactivity without impairing the host’s ability to guard against opportunistic infections, and malignancies, transplant patients who suffer new or recurrent malignancies often have no chance for another operation, and other treatment approaches may have no effect.

The feasibility of immunotherapy treatment for such patients is unclear. Although some patients have safely and effectively received checkpoint inhibitors therapy after kidney transplantation, and with the patients remaining on low-dose immunosupression during ipilimumab therapy and the closed monitor of kidney function, some of the patients experienced apparent benefits^22^–^24^, the immunotherapy clinical trails in OTRs are still very lacking and need much more investigations. Here, we summarize 3 cases of cancer immunotherapy in patients with new or recurrent tumors after liver transplantation.

## Cases in the literature

The first case^25^ was a 67-year-old man with a history of hepatitis C virus and HCC who underwent orthotopic liver transplantation 8 years ago. Three years after the transplantation, a biopsy in revealed an ulcerated melanoma at least 0.7 mm in Breslow thickness.And 4 years later, the HCC surveillance imaging revealed a 5 cm right adrenal mass that was subsequently resected, and the pathology revealed metastatic HCC. When finally the patient experienced multifocal disease progression in April 2014, a multidisciplinary team-based discussion including both medical oncology and transplant medicine, decided to begin the therapy on ipilimumab while maintaining rapamycin at 1 mg daily. Although he experienced a transient increase in aspartate aminotransferase or alanine aminotransferase levels >200 IU/L, his tumor shrinked. And when ipilimumab administration ended, the patient’s transaminases resolved to near baseline levels within 8 weeks.

In another case^26^, a 59-year-old female who underwent liver transplantation also for 8 years. She underwent excision of melanoma in her seventh year after transplantation; a follow-up chest computed tomography the next year revealed numerous bilateral pulmonary nodules that were confirmed to be metastatic lesions. She received 4 doses of ipilimumab, during which time serum tacrolimus concentration and liver function tests were checked weekly during treatment. In this patient, CTLA-4 inhibition did not induce graft rejection or immune adverse events. However, the patient did not show tumor shrinkage after treatment.

In the third case^27^, a 48-year-old man was diagnosed with lung metastasis at 4 months after a liver transplantation for primary HCC, so he took Sirolimus (2 mg/d) on the basis of Tacrolimus. Then the patient received the treatment of pembrolizumab (a single 150-mg intravenous infusion) at 12 months after transplantation in Hongkong, China. While liver dysfunction was found at the fifth day after the treatment; and a liver biopsy showed pathologic changes indicative of mild to moderate acute rejection. So the pembrolizumab treatment was paused. The patient was followed up for 8 months after that, and survived with tumor, but his liver function remained abnormal.

## Discussion

Although further study in a large patient cohort is warranted, these cases raise the question of the feasibility and efficacy in administering ipilimumab and other immunotherapy drugs to liver transplant recipients. Among those 3 patients, 2 tolerated immune checkpoint inhibitors without major adverse effects and at least 1 of the patients saw significant benefit, which imply that liver transplant recipients several years posttransplant may be appropriate candidates for trials with immunomodulatory treatments. Of course, strict laboratory and clinical monitoring are necessary to remain the values in a controllable range throughout treatment, only low dosing with immunosuppressives during ipilimumab therapy seems essential, as well as proper patient selection with the use of biomarkers. However there is no such biomarkers published so far.

Integrating immunotherapy into clinical care will pose challenges of its own. Just as chemotherapy comes with risks, so does ipilimumab (anti-CTLA-4). Side effects included fatigue, severe diarrhea, colitis, and endocrine disruption; 14 patients have died from this treatment^1^. And according to the reports in a series of 14 patients, the patients appeared hair repigmentation owing to anti-PD-1/anti-programmed death ligand 1 (PD-L1) therapy for lung cancer, and the authors believe that hair repigmentation may be a good response marker in patients receiving anti-PD-1/anti-PD-L1 therapy for lung cancer^28^. Some volunteers joining in the clinical trails of a kind of PD-1 drugs appeared the cherry hemangioma. The challenge now is to enhance the effectiveness of checkpoint inhibitors and while ameliorating adverse effects through combinations with chemotherapy, Tregs, or other agents that modulate cancer-immune cell interactions. Identifying which patients may tolerate both reduced immunosuppression and the use of immunomodulatory agents is critical. For example, the discrepancies in patient selection and PD-L1 testing methods should be the explanation of the contradictory results of recent phase III trials with immune checkpoint inhibitors in the first-line setting^29^. Although no definitive prognostic markers have been identified, several studies have shown increased length of time since transplantation to be a predictive factor for tolerance of immunosuppression withdrawal^30^,^31^. In addition, compared with other organs such as the heart, kidney and lung, liver grafts are considered to be the least immunogenic organs for transplant and thus can sustain less aggressive immunosuppressive regimens^32^.

Another issue is that patients may take months to respond, making it difficult to assess whether treatment is helping. Furthermore, some treatments are highly personalized and impossible to administer outside of specialized settings, which makes them extraordinarily expensive. In addition, the utility of reduction of immunosuppressive therapy and its relative contribution of the overall antitumor effect should be further investigated.

Many specialists wonder whether they can really become part of standard cancer therapeutic strategy. However, even doubters recognize that what is considered to be impossible in medicine is always changing. Monoclonal antibodies had the same stones thrown at them 20 years ago, with everyone questioning their feasibility. As Steven Rosenberg, a specialist in immunotherapy, said, “The goal right now is to find things that work, and when you find things that work, industry finds ways to make it happen”^1^. However, only 3 patients have been reported in the literature. This is both a small number but there is a strong possibility for publication bias, so people should be indeed careful. Overall the evidence does not support a go ahead for treating these patients with immunotherapy but clinical trials should be supported to increase our experience. Several challenges must be addressed to establish immunotherapy as a useful option for posttransplant patients with recurrent or new malignant tumors.

## Ethical approval

The program was approved by the Ethics Committee of The First Affiliated Hospital of Zhengzhou University.

## Sources of funding

Supported by the Scientific and Technological Project of Science and Technology Department of Henan Province (340600531842, 152300413242); Scientific and Technological Project of Medical Science and Technology Department of Henan Province (201401003).

## Author contribution

S.Z.: raised the idea and designed the project. W.G.: provided the cases in the literature. M.L.: wrote the manuscript with all authors contributing writing and providing feedback.

## Conflict of interest disclosure

The authors declare that they have no financial conflict of interest with regard to the content of this report.

## Research registration unique identifying number (UIN)

Not applicable.

## Guarantor

Shuijun Zhang.

## Acknowledgments

The authors are indebted to all members who helped them complete this study.

## Supplement

China has fully ceased the use of the death penalty prisoners’ organ for transplantation since January 1, 2015.
